# Synaptic-like coupling of macrophages to myofibers regulates muscle repair

**DOI:** 10.21203/rs.3.rs-5290399/v1

**Published:** 2024-10-28

**Authors:** Tripathi Gyanesh, Dourson Adam, Jennifer Wayland, Sahana Khanna, Megan Hoffmann, Thirupugal Govindarajan, Fabian Montecino Morales, Luis Queme, Douglas Millay, Michael P. Jankowski

**Affiliations:** Cincinnati Children’s Hospital Medical Center; Cincinnati Children’s Hospital Medical Center; Cincinnati Children’s Hospital Medical Center; Cincinnati Children’s Hospital Medical Center; Cincinnati Children’s Hospital Medical Center; Cincinnati Children’s Hospital Medical Center; Cincinnati Children’s Hospital Medical Center; Cincinnati Children’s Hospital Medical Center; Cincinnati Children’s Hospital Medical Center; Cincinnati Children’s Hospital Medical Center

## Abstract

Peripheral injury responses essential for muscle repair and nociception require complex interactions of target tissues, immune cells and primary sensory neurons. Nociceptors and myofibers both react robustly to signals generated from circulating immune cells, which promote repair, growth, and regeneration of muscle while simultaneously modulating peripheral sensitization. Here, we found that macrophages form a synaptic-like contact with myofibers to hasten repair after acute incision injury and to facilitate regeneration after major muscle damage. Transient chemogenetic activation of macrophages enhanced calcium dependent membrane repair, induced muscle calcium waves *in vivo,* elicited low level electrical activity in the muscles and enhanced myonuclear accretion. Under severe injury, macrophage activation could also modulate pain-like behaviors. This study identifies a novel mechanism by which synaptic-like functions of macrophages impacts muscle repair after tissue damage.

## Introduction

One mechanism for skeletal muscle injury is through disruption of the integrity of the myofiber plasma membrane and basal lamina, leading to altered intracellular calcium levels [1, 2]. Functional restoration after muscle damage requires a coordinated response of multiple cellular systems including the myofibers, immune cells and sensory neurons [3]. Muscle recovers through a variety of mechanisms that include restoring the disrupted basal lamina and myofiber membrane, or if necessary, myogenesis, ultimately resulting in re-establishment of muscle function [4, 5]. Importantly, to allow proper repair of peripheral structures, an indispensable nociceptive signal is generated to inform the organism of the ongoing repair process [6]. Nociceptors and myofibers both respond robustly to signals generated from circulating immune cells [7] [8]. Given the architecture of skeletal muscle tissue and the wide range of injuries and diseases that prompt muscles to regenerate, it is important to obtain a better understanding of the time-course required and multi-cellular interactions that are established during various injuries to facilitate muscle repair [4].

Immune cells, including macrophages, infiltrate sites of injury to facilitate inflammation and repair processes [9–11]. In muscle, they can promote myofiber growth and regeneration [12, 13]. Macrophages release a variety of molecules including cytokines and chemokines which can play a role in both muscle repair and nociception [14–16]. Vesicles are used to package these factors within macrophages and can contain other molecules such as various proteins or ions that are concurrently released with the cytokines/chemokines [17, 18]. A recent study showed that in the heart, resident macrophages could also electrically couple to cardiomyocytes to modulate their function through delivery of calcium ions between cells [19]. It has further been shown that macrophages can transport mitochondria to sensory neurons in the DRGs [20] under injury conditions suggesting direct connections may be possible with neurons as well. However, it is not known whether a similar phenomenon with circulating immune cells could dually modulate skeletal muscle repair and/or nociception after injury. A better understanding of the complex interactions between these cellular systems would fill a major gap in our understanding of myalgia development and/or disorders of muscle repair/function. In this study, we hypothesized that infiltrating macrophages would couple to both myofibers and neurons to dually modulate muscle repair and pain. We performed a variety of assays assessing muscle repair and pain after acute or severe injury with chemogenetic or optogenetic manipulation of infiltrating macrophages and compared results to mice with synaptic transmission inhibited in macrophages. We found that macrophages form synaptic-like contacts with myofibers to hasten repair after both acute traumatic injury and major muscle damage. Interestingly however, muscle repair could be dissociated from pain-like behaviors during acute injuries indicating that these two distinct biological processes can be distinguished at the level of the infiltrating macrophage.

## Results

### Chemogenetic activation of macrophages restores membrane integrity after acute incision injury.

**Figures 1:**
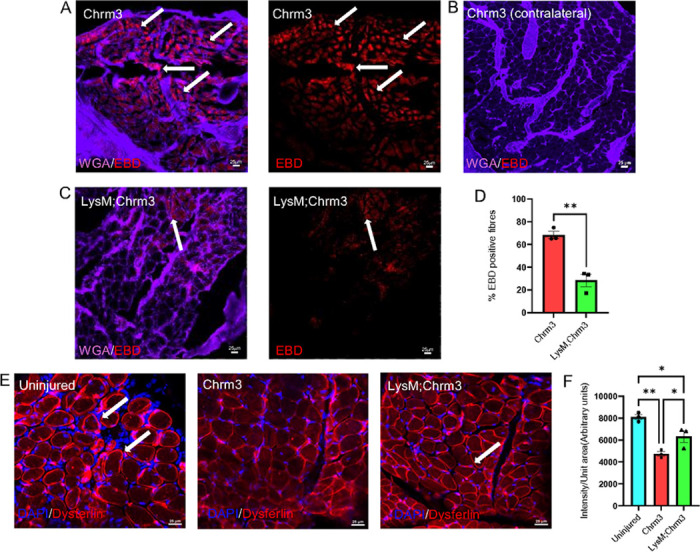
Transient chemogenetic activation of macrophages regulate muscle repair. EBD (red) is readily detectable in muscle in control (Chrm3) mice treated with CNO + incision at 2d (A) compared to contralateral control muscle (B), but this is inhibited in LysM;Chrm3 mice (C) with CNO+Incision. Quantification of EBD+ myofibers in the indicated groups (D). Purple=WGA; **p<0.01 vs. Chrm3. Unpaired t-test, n=3 mean±SEM. Dysferlin (red) is readily detectable in muscle in uninjured muscles, and this is reduced at 2d post incision in control mice. CNO treatment in LysM;Chrm3 mice with incision reverses this reduction (E). Quantification of dysferlin intensity in our groups (F). DAPI=blue; *p<0.05 vs. uninjured. ***p<0.005 vs uninjured and Chrm3+Inc. 1-way ANOVA with Tukey’s post hoc, n=3, mean ± SEM, Scale Bars, 25mm.

Reports suggested that macrophages may be able to couple to other cell types to modulate their functions [21, 22]. We therefore utilized a chemogenetic strategy to induce calcium signaling selectively but transiently within macrophages (LysMCre;Chrm3 (GqDREADD)) in mice with incision injury to the flexor digitorum brevis (FDB) muscle. To tailor macrophage activation to shortened time frames, we only injected mice with CNO 1x daily beginning 1d after incision injury and immediately (within 1hr) prior to behavioral analyses. To first determine if transient activation of macrophages could modulate muscle repair after injury, we assessed Evan’s Blue Dye (EBD) uptake in mice with incision. We found that incision of the muscles increased EBD uptake in the myofibers 2d after incision compared to uninjured contralateral muscles (Figs. 1A-B). Surprisingly, we found that transient chemogenetic activation of macrophages was able to significantly block the uptake of EBD into the muscles and partially restore membrane integrity after incision (Fig. 1C-D). To confirm that calcium related repair processes could be at play in the observed hastening of myofiber repair after incision injury, we isolated muscles from our CNO treated LysM;Chrm3 mice with incision injury on day 2 and stained muscles for the calcium dependent membrane repair protein, dysferlin. We found that incision-related reduction in dysferlin staining was restored by chemogenetic activation of macrophages in mice with incision (Fig. 1D-E). Validation of calcium activation in macrophages containing the GqDREADD were performed *in vitro* and demonstrated that CNO can effectively induce calcium production in these immune cells (Supplementary Fig. 1). This suggests that transiently activating calcium in macrophages can facilitate myofiber repair after injury and the calcium dependent repair processes rely on macrophage signaling to muscles.

To then assess whether transient activation of macrophages could alter pain-like behaviors in mice with incision, we performed spontaneous and evoked assessments of hypersensitivity in our LysMCre;Chrm3 mice treated with CNO. Surprisingly, we found that incision provoked spontaneous paw guarding and reduced muscle withdrawal thresholds by 1d after incision but transient chemogenetic manipulation of macrophages did not alter any of these behavioral changes induced by incision at any time point (Supplementary Fig. 2). This suggests that muscle repair processes can be dissociated from pain-like behaviors by the way macrophages interact with myofibers after incision injury.

### Activation of macrophages induces calcium transients in muscle tissue and rapidly modulates electrical activity in the muscles.

**Figure 2:**
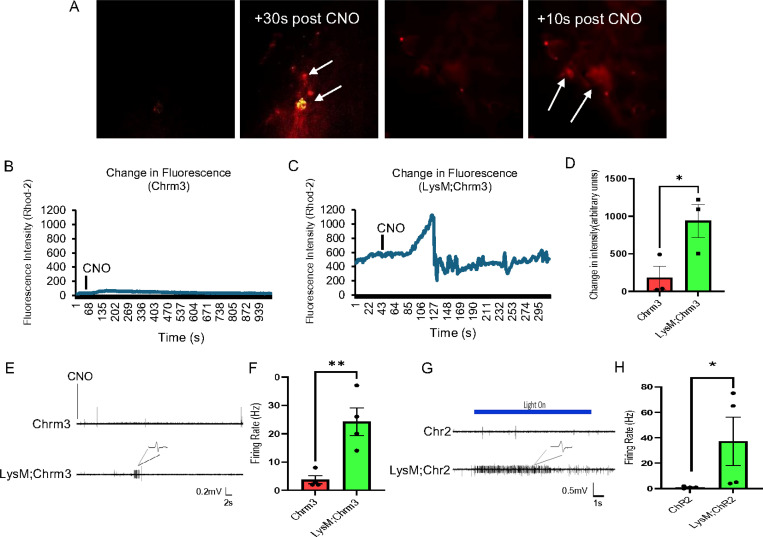
Transient activation of macrophages induces calcium transients in muscle. Example images of calcium induction (Rhod-2, red, arrows) in muscle *in vivo* in LysM;Chrm3 mice before (left) and 10–30s after (right) delivery of CNO to the hind paw (A). Example traces of fluorescence intensity changes in muscle upon CNO delivery in control (B) and LysM;Chrm3 (C) mice with incision. Quantification of changes in peak fluorescence between conditions (D). *p<0.05 vs Chrm3, Unpaired t-test, n=3, mean±SEM. Chemogenetic activation of macrophages increases EMG activity in the muscles of LysM;Chrm3 mice compared to Chrm3 controls (E). Firing rates are significantly increased in the LysM;Chrm3 mice (F). **p<0.01 vs Chmr3. Unpaired t-test, n=4. Similar results are obtained in mice with optogenetic activation of macrophages. Immediately upon blue light delivery, EMG activity is detected in muscles in LysM;Chr2 mice with incision (G). This is not detected in controls (Chr2). Firing rates are significantly increased in the LysM;Chr2 mice (H). *p<0.05 vs Chr2. Mann-Whitney test, n=4, mean ± SEM.

We then wanted to assess how DREADD dependent activation of macrophages may modulate muscle function. We therefore performed electromyographic (EMG) recordings in LysMCre;Chrm3 mice with incision and compared to controls. Similar to intravital calcium imaging experiments, CNO was delivered directly to the hind paw during EMG recording. Interestingly, within a relatively short time frame after CNO delivery (~ 10–30s), we detected bursts of activity within the muscles of LysMCre;Chrm3 mice with incision, but not in the Cre negative incised animals (Fig. 2E-F). This suggests a direct correlation between calcium activation in macrophages and electrical activity in the muscles.

Although results were promising and indicated that macrophage activation can directly and rapidly regulate muscle function during incision, we could not confirm whether this was due to slow release of vesicles from macrophages onto the muscle fibers or whether a more direct delivery was established. To begin to determine this, we performed electromyography on hind paw muscles from incised mice with channelrhodopsin expressed in macrophages (LysMCre;Chr2). Surprisingly, we found an almost immediate and prolonged induction of EMG activity upon delivery of blue light to the muscles that was not observed in Cre negative controls with incision (Fig. 2G-H). These data strongly support a direct coupling mechanism between macrophages and muscle fibers that modulates muscle function and repair processes after injury.

### Macrophage activation enhances muscle regeneration and pain-like behaviors after cardiotoxin injury.

**Figure 3:**
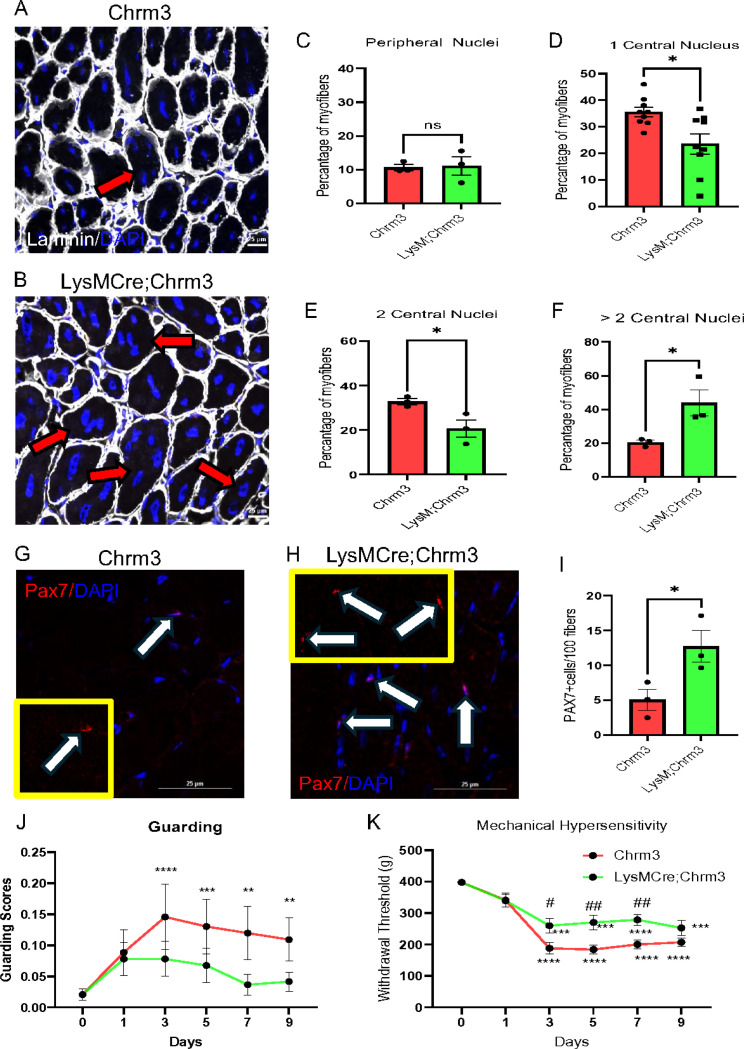
Chemogenetic activation of macrophages alters muscle regeneration. 10d after cardiotoxin (CTX) injection into the tibialis anterior muscle of control mice treated with CNO (Chrm3), many myofibers (indicated by red arrows) surrounded by laminin (white) display central nuclei (blue; A). In LysMCre;Chrm3 mice treated with CNO after CTX injection, more fibers show multiple nuclei (B). Quantification of peripheral, central and multinucleated myofibers in our groups at 10d (C-F), unpaired t-test, n=3, mean±SEM. Control Chrm3 mice injected with CTX also show some pax7 positive cells (red) in the muscles (white arrows) at 10d (G). CNO treated LysM;Chrm3 mice with CTX at 10d however display greater pax7 staining (arrows) in the muscles (H). Quantification of pax7 positive cells per 100 myofibers in CNO treated Chrm3 and LysM;Chrm3 mice injected with CTX at 10d, unpaired t-test, n=3, mean±SEM, Scale Bars, 25mm. (I). Control (Chrm3) mice display significant guarding (J) and mechanical hypersensitivity to muscle squeezing (K) beginning 3d post CTX injection. These CTX-induced behaviors are significantly inhibited in LysMCre;Chrm3 mice treated with CNO. **p<0.01, ****p<0.001,***p<0.0005 vs baseline, #p<0.05, ##p<0.01 vs time matched controls. 2-way RM ANOVA with Tukey’s post hoc, n=16, mean ± SEM.

As there appeared to be direct coupling of macrophages to muscle tissue after acute traumatic injuries to regulate repair, we wanted to also determine if this phenomenon could be observed in other models of muscle injury. We thus performed an analysis of muscle regeneration from LysMCre;Chrm3 mice injected with cardiotoxin (CTX) into the tibialis anterior muscles which causes significant disruption of the muscles and allows for the assessment of muscle regeneration. Quantification of peripheral and central nuclei per myofiber in addition to fibers with more than 2 nuclei were determined in our groups. We found that in control mice with CTX injury, we observed the expected increase in central nuclei present per myofiber, 10 days post injection. However, in LysMCre;Chrm3 mice injected with CTX and then CNO every other day beginning at 1d, we found an increase in fibers with more than 2 nuclei indicating either a hastening or enhanced level of muscle regeneration post CTX (Fig. 3A-F). No differences in peripheral or central nuclei were found between our groups at 3d or 7d post CTX injection (Supplementary Figs. 3–4). To further characterize the effects of activating Gq in macrophages on muscle regeneration, we assessed stem cell activation marker, Pax7, over time post CTX injection in our groups. Although no changes in Pax7 cells were detected at 3d or 7d post CTX between groups (Supplementary Figs. 3–4), we did observe a significant increase in Pax7 cells in the muscles from LysMCre;Chrm3 mice with CNO delivery at 10d post CTX injection (Fig. 3G-I). To determine if any pain-related behaviors could be modified in the CTX model, we performed similar guarding and mechanical hypersensitivity measurements in CNO treated LysMCre;Chrm3 mice injected with CTX. We found that control animals with CTX show modest guarding behaviors and a delayed onset mechanical hypersensitivity to muscle squeezing that lasted for the duration of our testing period (10d). Surprisingly, unlike that observed with incision injury (see Supplementary Fig. 2), LysMCre;Chrm3 mice injected with CTX and then CNO every other day display reduced guarding and mechanical hypersensitivity (Fig. 3J-K). These data indicate that macrophage activation can enhance muscle regeneration in severe injury models and under these larger injury conditions, activation of these immune cells can also modulate pain-related behaviors over time.

### Macrophages make synaptic-like contacts with muscles to modulate repair.

As our data thus far suggested a rapid regulation of muscle repair upon activation of macrophages, we wanted to assess how this may occur. Macrophages are known to release a variety of cytokines and chemokines to modulate inflammation and repair [9, 22]. We therefore assessed the levels of select cytokines and chemokines in the muscles of our LysMCre;Chrm3 mice with incision or CTX injection and treated with CNO. Interestingly, in mice with incision injury, no changes in genes such as IL6, TNFa, IL1b or MCP1 were observed in CNO treated LysMCre;Chmr3 mice compared to treated Chmr3 controls at 2d post injury. We observed similar effects in the CTX treated mice with CNO for TNFa, IL1b and MCP1 but we did observe an increase in IL6 in both LysMCre;Chrm3 mice and Chrm3 controls at 10d post CTX (Supplementary Fig. 5). Data suggests that transient activation of macrophages *in vivo* has minimal effects on production of cytokines/chemokines after incision or CTX injury.

**Figure 4:**
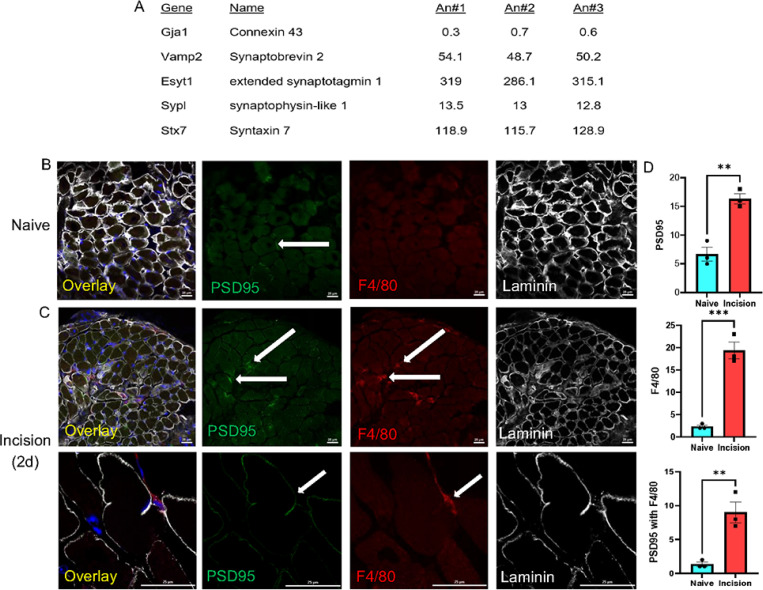
LysM+ macrophage RNA-Seq and PSD95 staining in muscles from mice with incision. RNA-Seq analysis for indicated genes from sorted LysMCre;tdTomato+ cells (A). Although gap junction mRNAs such as Gja1 (i.e. connexin 43) are not readily expressed in macrophages (TMP values need to greater than 10 for reliable detection), synaptic proteins are highly expressed in macrophages. An = animal. Post-synaptic density protein 95 (PSD95, green) is detectable in muscle marked by laminin (white) in naïve mice (B). Increased PSD95 is detected 2d post incision in the muscles and these are often colocalized with macrophages (F4/80, red, C). Quantification of PSD95 and F4/80 in muscles from mice with incision indicates an increase in PSD95 that colocalizes with F4/80+ macrophages (D). **p<0.01, ***p<0.001 vs naïve. Unpaired t-test, n=3 mean ± SEM, Scale Bars, 25mm.

Since previous reports suggested that resident macrophages can form gap junctions with cardiomyocytes to regulate their function [19] it is plausible that circulating macrophages formed similar contacts in our conditions. We therefore first wanted to verify that our infiltrating macrophage population contained appropriate proteins for generation of gap junctions which could serve as “electrical synapses” between macrophages and myofibers. We analyzed our previously obtained RNA-Seq dataset on macrophages sorted using fluorescence activated cell sorting (FACS) methods obtained from macrophage reporter mice (LysMCre;tdTom) [23]. Surprisingly, we found that LysM + cells do not contain gap junction proteins. They do, however, express high levels of synaptic proteins including synaptobrevin 2 (VAMP2), extended synaptotagmin 1 (Esyt1), synaptophysin-like 1 (Sypl) and syntaxin 7 (Stx7) to name a few (Fig. 4A).

We therefore wanted to determine if infiltrating macrophages were forming synaptic-like contacts with myofibers instead of forming electrical synapses using gap junction proteins. Muscle tissues were thus stained from mice with incision injury for the synaptic marker PSD95 along with laminin and F4/80 (another macrophage marker). An increase in PSD95 + contacts were observed in mice with incision injury that co-localized with apposed F4/80 + macrophages (Fig. 4B-D). These results with those presented above collectively indicate that macrophages may be generating *synaptic-like* contacts to modulate repair.

**Figure 5:**
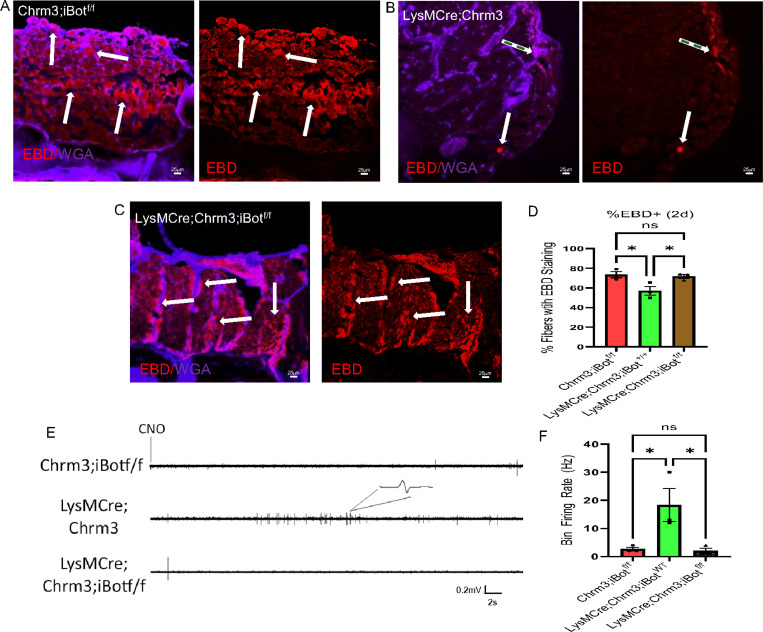
Blocking synaptic vesicle release inhibited the effects of chemogenetically activating macrophages onmuscle repair. Examples of EBD labeling from Cre negative, Chrm3;iBotf/f control (A), LysMCre;Chrm3 (B) and LysMCre;Chrm3;iBotf/f (C) groups 2d after incision injury to the FDB muscles. Quantification of EBD positive fibers per section is provided (D). *p<0.05 vs indicated condition. 1-way ANOVA with Tukey’s post hoc test. LysM;Chrm3 mice containing Cre inducible botulinum toxin in macrophages display little EMG activity upon CNO delivery unlike that observed in LysM;Chrm3 mice *in vivo* (E). Quantification of firing rates post CNO in three groups listed (F). *p<0.05, 1-way ANOVA with Tukey’s post hoc test, n=3, mean ± SEM. Scale Bars, 25mm.

To test this idea, we crossed LysMCre;Chrm3 mice with a Cre-inducible botulinum toxin mouse (iBot), which will disrupt synaptic machinery, including VAMP2 [24]. In these mice, macrophages will not be able to release vesicles via these synaptic protein complexes even after chemogenetic activation of intracellular calcium using the GqDREADDs. We therefore performed EBD uptake and EMG analyses on muscles from our groups and found that LysMCre;Chrm3;iBot^f/f^ mice treated with CNO after incision display increased EBD in the muscles 2d post incision and have reduced EMG activity (Fig. 5) similar to mice with no GqDREADD expressed in macrophages at this time point. To finally confirm if this was calcium dependent release from macrophages, we performed calcium imaging experiments *in vitro* on macrophages isolated from the LysMCre;Chmr3 mice and treated cultures with CNO. Cells were grown in calcium free media to observe any release of these ions into the bath upon CNO treatment. We indeed found that chemogenetic activation of macrophages caused calcium to be detected in the bath readily upon CNO treatment while Cre negative macrophages showed little changes in bath levels of calcium (Supplementary Fig. 6). Together these results indicate that infiltrating macrophages may synaptically couple to myofibers after damage to facilitate a calcium dependent repair process in the muscles.

## Discussion

This report is the first to show that infiltrating macrophages can form synaptic-like contacts with myofibers (Figs. 4–5) to rapidly regulate (Fig. 2) calcium-dependent repair in the muscle tissue (Figs. 1,3). After severe muscle injury, transient macrophage activation could also modulate pain-like behaviors (Fig. 3), however, during acute injuries, pain could be dissociated from muscle repair when macrophages were briefly activated (Supplementary Fig. 2). Results uncover a novel mechanism by which circulating immune cells contribute to repair processes in the skeletal muscles after damage.

Muscle injuries occur from a variety of causes, including direct trauma (e.g., lacerations, contusions, or strains) or indirect causes (e.g., ischemia or neuromuscular dysfunction) [25, 26]. The general phases of healing occurring within the damaged muscle are mostly similar among the various types of muscle injuries, but the functional recovery of the injured muscle varies from one type of injury to another. It has become clear that the processes occurring in injured muscle (i.e., necrosis/degeneration, inflammation, repair, and scar-tissue formation [fibrosis]) are all interrelated and time-dependent [1, 27, 28]. In this study we considered hind paw incision injury as an acute trauma model [23, 29, 30] and cardiotoxin injury [31–33] as major injury condition resulting in muscle degradation. Skeletal muscle repair is a highly synchronized process involving the activation of various cellular and molecular responses, where the coordination between inflammation and regeneration is crucial for the beneficial outcome of the repair process following muscle damage [34, 35].

Data from several models of muscle injury (hindlimb ischemia, freeze-injury, unloading/reloading, and myotoxic agent injections) indicate that impairment of immune cell recruitment including macrophages into injured muscle results in delayed tissue regeneration in terms of appearance of regenerating central nucleated myofibers and persistence of intramuscular adipocytes and fibrosis [36–38]. Part of the contribution of macrophages to repair processes relies on their removal of debris after injury. However, several findings suggest that macrophages may play a more direct role in muscle repair and remodeling than merely removing tissue debris. For example, muscle regeneration by transplanted myogenic cells can be impaired if the recipients of whole muscle grafts are depleted of monocytes and macrophages by irradiation before transplantation, which has been interpreted as showing a role for macrophages in muscle repair and regeneration *in vivo* [39].

A unique feature of infiltrating immune cells is that in addition to releasing cytokines and chemokines [40, 41], they may physically couple to tissues through connexin 43 (Cx43) dependent gap junctions [42]. A recent report suggests that resident macrophages in particular have the capacity to *electrically* couple to cardiomyocytes in the heart and modulate atrio-ventricular (AV) conduction via newly formed Cx43 gap junctions [19]. Interestingly, peripheral nociceptors can also form new Cx43 gap junctions with each other in the dorsal root ganglion (DRG) after injury to regulate nociceptive responses [43–45]. Thus, it was reasonable to hypothesize that *infiltrating* immune cells such as macrophages could have electrically coupled to damaged myofibers to facilitate functional recovery [40, 46]. However, we found that infiltrating macrophages did not contain gap junction proteins but highly expressed synaptic-like proteins (Fig. 4). This effectively permitted these circulating cells to rapidly communicate with muscle (Fig. 2) without tissue to modulate both calcium transients in myofibers and EMG activity in the muscles altering pain-like behaviors during acute muscle injury (Supplementary Fig. 2).

Evan’s blue dye, considered as a marker to study muscle fiber integrity [47, 48], was used here to show that membrane disruption after injury could be restored in a hastened time frame upon chemogenetic activation of macrophages (Fig. 1). To confirm that calcium related repair processes were at play in the observed phenomena we stained muscles from our conditions with dysferlin which is calcium dependent membrane repair protein [49, 50]. Complementing EBD studies, dysferlin levels were also found to be restored upon chemogenetic activation of macrophages in mice with incision injury (Fig. 1). Moreover, to check the effects of macrophage activation on muscle tissue, calcium imaging and EMG recordings were performed. Not only did chemogenetic activation of macrophages induce calcium transients in muscle *in vivo* and in myotubes *in vitro*, induction of calcium signaling in these cells also modulated EMG activity in the muscles (Fig. 2). Although these studies indicated a direct effect of macrophages on muscle tissue, the possibility of rapid vesicle release to modulate repair remained [51]. We therefore performed optogenetic activation of macrophages during EMG recordings and observed an immediate onset of EMG activity in the muscle during light dependent activation of macrophages (Fig. 2). The amplitudes of the EMG waves were small in most cases both in the chemogenetic and optogenetic experiments, but in other cases were nevertheless seemingly capable of inducing a contraction (Supplementary Videos). Nevertheless, these experiments suggest that regardless of a direct behavioral effect, transient activation of macrophages was able to rapidly initiate low level EMG activity that also could facilitate calcium dependent muscle repair and regeneration (Figs. 1–3). There is obviously still a role for released factors from macrophages in the inflammatory and repair processes [9], but this work also uncovers a novel mechanism by which macrophages can modulate repair.

We were able to confirm increases in synaptic-like contacts between macrophages and skeletal muscles after incision, that infiltrating macrophages do contain synaptic proteins, and inhibition of synaptic-like transmission in macrophages blocked the chemogenetic effects of macrophages on myofiber repair and EMG activity (Figs. 4–5). Thus, even though *electrical* coupling was not confirmed as was shown in the heart [19], synaptic-like coupling was observed between infiltrating macrophages and myofibers to regulate repair after incision. Specifically, postsynaptic density protein (PSD95) was colocalized with macrophages [52, 53]. This is the most abundant protein located at excitatory synapses in the nervous system and is involved in the stabilization, recruitment and trafficking of various receptors to the postsynaptic membrane [54–56] and serves as a major component of neuromuscular junction (NMJ). Our data now includes a novel mechanism by which synapse-like contacts form between immune cells and myofibers to regulate repair.

We further showed a role for these novel contacts between macrophages and muscle in more severe injury models like CTX injury. Similar to that found after incision, activation of macrophages could hasten regeneration after damage (Fig. 3) [57–59]. Normal skeletal muscles are composed of individual multinucleated myofibers with nuclei positioned at their periphery. Interestingly, some nuclei are positioned in the center of the myofiber, which is a marker of myofiber repair and has long been recognized as a sign of diseased muscle tissue [59] [60]. In our severe injury model, LysMCre;Chrm3 mice treated with CNO injection after injury showed more fibers with multiple nuclei and increased pax7 staining suggesting faster recovery than the control mice. Muscle injury typically initiates a rapid and sequential infiltration of muscle by inflammatory cell populations that can persist for hours to weeks, while muscle repair, regeneration, and growth occur [15]. This relationship between inflammation and muscle repair or regeneration has suggested that muscle inflammation after modified muscle use may be functionally beneficial.

Another interesting finding from these studies is the fact that pain-like behaviors were dissociated from tissue repair processes under acute injury conditions such as incision. This indicates that once initiated, sensitization mechanisms in neurons may continue their normal trajectory of sensitization and resolution regardless of the state of tissue repair. This would challenge the traditional dogma that pain manifests alongside tissue injury and resolves upon repair. We clearly showed here that through transient infiltrating macrophage activation, we can hasten muscle repair after incision injury at a time point when animals still display acute pain-like behaviors (Fig. 1, Supplementary Fig. 2). Future experiments analyzing this relationship are therefore crucial. Nevertheless, these studies will offer a novel, mechanistic understanding of nociceptive processing and muscle repair after tissue damage in relation to the synaptic-like functions of macrophages. These studies are uniquely positioned to dramatically advance our mechanistic understanding of muscle pain development and myofiber repair and could identify a novel target for therapeutic intervention in several muscle injury states.

Mendeley Data Repository Link to Data and Videos: https://data.mendeley.com/drafts/4zbzb3rfg3

## Materials and Methods

### Animals

Experiments were conducted with young adult male and female mice. Several transgenic lines were used in various breeding pairs. We used the following lines in the current report: LysM-Cre (Jax stock #004781), Rosa26-LSL-hM3Dq-mCit (Jax #026220), Rosa26-LSL-Chr2 (Jax #024109), Rosa26-LSL-iBot (Jax #018056). Mice were bred to generate LysMCre;Chrm3, LysMCre;Chr2 and LysMCre;Chrm3;iBot mice along with their littermate controls. All animals were housed in a barrier facility in group cages of no more than four mice, maintained on a 14/10 hour light/dark cycle with a temperature-controlled environment, and given food and water ad-libitum. All procedures were approved by the Cincinnati Children’s Hospital Medical Center Institutional Animal Care and Use Committee and adhered to National Institutes of Health Standards of Animal Care and Use under American Association for Accreditation of Laboratory Animal Care-approved practices. Animals were anesthetized with 2–3% isoflurane before induction of various treatments or with ketamine/xylazine (100 mg/kg / 10 mg/kg) prior to terminal procedures.

### Hind paw Incision and Cardiotoxin (CTX) injury

Mice were anesthetized with 2–3% isoflurane and then an incision was performed from the hairy skin side of the hind paw between the bones through to the flexor digitorum brevis muscles. Blunt manipulation of the muscle was performed using #5 forceps, but the plantar skin was left untouched. Wounds closed with 6–0 sutures [7].

For CTX (Millipore Sigma, 217503) experiments (CTX aliquoted in sterile saline at a concentration of 10 μM), the agent was delivered directly into the tibialis anterior muscles. Mice were first anesthetized with isoflurane and legs shaved. Then 50 μL of CTX was injected using a 28-gauge needle [61].

### Pain-related Quantitative Behavioral Paradigms

Behavioral examination of spontaneous paw guarding, and mechanical withdrawal thresholds using muscle squeezing assays were performed as described previously [62]. All behavioral assays were conducted by experimenters blind to conditions.

### Spontaneous paw guarding

Before the commencement of behavioral studies, mice were randomly assigned to different groups into a rectangular transparent plastic box where they were acclimatized for approximately 10 minutes. Baseline pain scores were measured before the induction of injury. A cumulative pain score was used to assess spontaneous pain like behavior using a rating scale developed by Xu and Brennan [62]. Mice were allowed to move freely inside the transparent plastic box. The ipsilateral and the contralateral paws were closely monitored during a 1-minute period repeated every 5 minutes for 60 minutes. The testing scale scores movement of the hind-limb from where there is no observable hind paw movement off of the floor of the box (score = 0), through when a slight raise is observed (score = 1) to when the paw is completely raised off of the floor (score = 2). Guarding was evaluated one animal at a time at 5-minute intervals for twelve readings and the average score is used as the mean value.

### Mechanical withdrawal thresholds

The modified paw pressure device was used to quantify the nociceptive withdrawal reflex after muscle injury. This device was used to apply increasing mechanical force (up to 350 gram) on the plantar surface of both hind paws using a blunt/rounded tip which allows for stimulation of the muscles until a paw withdrawal is observed. The force at which the paw withdrew was determined to be threshold and this was repeated three times with a 5-minute interval in between trials. The average of the three measurements was determined per animal and measurements were averaged across groups for comparisons. For LysMCre;Chr2 mice, the hind paws were stimulated with blue light for 10 seconds (10mW) to assess spontaneous withdrawal and then immediately after optogenetic stimulation, withdrawal threshold were performed as mentioned above using von frey filaments.

### Immunocytochemistry

In order to evaluate the integrity of the skeletal muscle fibers, we injected mice (i.p.) with 200 μl of Evans-blue dye (EBD: 1% in 0.9% sterile saline solution). EBD has been used extensively to evaluate the integrity and permeability of the membrane of muscle fibers [63]. Wheat germ agglutinin (WGA) conjugated with FITC (Life Technologies) was used to co-stain the tissue to visualize the membranes in the skeletal muscle, as previously described [64]. Briefly, muscle tissue was embedded in Tissue-Tek O.C.T. compound (Sakura Finetek USA Inc.), flash frozen in liquid nitrogen and sectioned at 12 μM on a cryostat and mounted on slides. Tissue was fixed on slide using 4% paraformaldehyde in 0.1 M PBS. The samples were subsequently washed, blocked in 0.01 M PBS containing 5% horse serum, 1% bovine serum albumin, and 0.2% Triton X-100 for 10 min. Sections were stained with WGA-FITC (1:100), incubated for 1 h, washed and cover slipped.

In other experiments, muscle samples were collected from LysM;Chrm3 and control mice in the same way and stained with dysferlin antibodies (rabbit anti-dysferlin 1:200; Abcam, catalog #ab124684) using an overnight incubation. Sections were then washed with PBS and incubated with labeled secondary antibodies (Alexa Fluor 488, dilution 1:400; Thermo Scientific, catalog# A11034) for 35 mins at room temperature and cover slipped after PBS washing.

A separate set of muscle samples from mice were cut and stained with PSD95 (rabbit anti PSD95 1:1000 Gentex, catalog # GTX133091), F4/80 (Rat anti F4/80 1:200 Abcam, catalog #ab 6640), and Laminin (rabbit anti Laminin 1: 200 Abcam, catalog #ab 15277), incubated overnight, washed and labeled with appropriate secondary antibodies (Alexa Fluor Fab 488, Jackson ImmunoResearch, catalog # 111–547-003), 594 Donkey anti Rat, Thermo Scientific, Catalog # A21209), 647 goat Anti Rabbit, Thermo Scientific, A21244) at the dilution of 1:400 respectively for 35 mins and cover slipped after PBS washing.

In order to quantify nuclei in the CTX treated mice, muscle samples were collected as mentioned above after 3, 7 and10 days of CTX injection which were stained with laminin (rabbit anti Laminin 1: 200, Abcam, catalog #ab 15277), incubated overnight, washed and labeled with secondary antibody 647 goat Anti-Rabbit(Thermo Scientific, catalog # A21244) for 35 mins. The slides were then rinsed in PBS and cover slipped using Fluro media with DAPI (Electron microscope Sciences, Cat# 17985–50) to stain nuclei. Nuclei were determined in three non-consecutive sections and quantified as containing either one, two or multiple nuclei per myofiber and reported as mean ± SEM.

For anti- Pax7 staining an additional antigen retrieval step was included in addition to above protocol, boiling slides in 1× Antigen Retrieval Citra Plus Solution (Invitrogen,# 00500) for 30 minutes before blocking with M.O.M. mouse IgG blocking reagent (MKB-2213-NB),followed by incubation with 1:10 dilution Pax 7 antibodies (DSHB, #AB528428 ) overnight. The following day, secondary (IgG1) (Thermo scientific, 555, A-21127) was incubated for 30 min similar to that described above followed by cover slipping.

Distribution of fluorescent staining was determined with a Nikon confocal microscope with sequential scanning to avoid bleed-through of the fluorophores. Three nonconsecutive sections, separated at least by four sections, from three different animals per condition were used to quantify the images. Exposure times during microscopic analysis for each image was performed at the same intensity level to confirm staining above background. All the muscle cells in a section were labeled using ImageJ and muscle cells that were observed to contain red staining were considered positive. The percentage of positive cells obtained from each animal was used for comparisons.

### Intravital calcium imaging

Mice were anesthetized under isoflurane anesthesia, had the hind paw muscles exposed and the hind paw isolated in a clay mold to allow superfusion of Krebs-Heinslfelt buffer (KH buffer) with 5% CO_2_ for 10 mins. The hind paw muscles were then loaded with 5% Rhodamine-2 AM dye in KH buffer, for 30 mins before imaging. Muscles of the anesthetized mice were then analyzed under a two-photon confocal microscope (Nikon FN1 upright Mutliphoton) after rinsing with KH buffer for 2–3mins. CNO (2mL of 0.4 mg/ml) was delivered directly to the hind paw during imaging to assess changes in calcium transients upon chemogenetic activation of macrophages. Videos were taken for up to 5 minutes. 3 random ROIs (region of interest) at red emission filter (around 580nm) using NIS software were chosen from each condition for time dependent analysis of fluorescence intensity. Changes in fluorescence intensity were then analyzed and the change in intensity was calculated as DF/F=(Fmax-FO)/FO. Fmax = maximum intensity during stimulation. FO = intensity measured in the ROI immediately prior to delivery of CNO. Data was averaged within a condition and compared across indicated conditions.

#### In vitro calcium imaging

For macrophage cultures analyzed in calcium free media, macrophages from LysMCre;Chrm3 and control mice were collected from peritoneal cavity by injecting isolation media (3–5 mL of cold sterile PBS with 3%FBS) into the peritoneal cavity of mice. Subsequently, the cell-containing fluid of the peritoneal cavity and isolation media was gently collected in falcon tube, then centrifuged and resuspended in complete MEM-alpha medium (Sigma, M4655–1). After 24 hours, non-adherent cells were discarded and replaced with fresh medium. This allows for isolation of peritoneal macrophages as > 90% of cells remaining in dish will be macrophages. For imaging the following day, wells were loaded with 5% Rhodamine-2 AM dye in KH buffer (without calcium component) for 30 mins at 37°C/5% CO_2_ and washed with KH buffer. 100ml of CNO was delivered directly to the wells during imaging to assess changes in calcium transients upon chemogenetic activation of macrophages. During analysis, macrophages were encircled as ROIs (region of interest) at red emission filter (around 580nm) using NIS software for time dependent analysis of fluorescence intensity. Changes in fluorescence intensity were then analyzed, average of the cells per well were calculated across each condition, then the average of the three wells were determined per condition and the change in intensity was calculated for that group and reported. Specific cell numbers are indicated in the figure legends for the respective experiment.

### Electromyography (EMG) on hind paw muscle

Electromyography was performed as previously described [65]. Briefly, mice were first anesthetized with 2% isoflurane. The sciatic nerve was exposed near the biceps femoris muscle. Then the hind paw muscles including the flexor digitorum brevis muscles were exposed. Mylar-coated steel recording wires (California Fine Wire) were implanted into the flexor digitorum brevis muscles, and reference wires were inserted under the skin near the base of the tail. A concentric bipolar stimulating electrode was placed on the sciatic nerve and used for electrical activation to confirm connectivity.

CMAPs were amplified using an Axoclamp 900a, recorded with a Micro 1401 data acquisition unit, and analyzed offline with Spike2 software (Cambridge Electronic Design, Cambridge, UK). A 2mA electrical stimulation of the sciatic nerve immediately proximal to the tibial, sural, and common peroneal branches was employed via a stimulus isolation unit (World Precession Instruments) connected to the Micro 1401. In the indicated instance, either blue light (10mW) or CNO was delivered directly to the hind paw muscles. Activity was recorded for 2min. After recording, the sciatic nerve was axotomized. The proximal end of the sciatic nerve was stimulated to ensure that CMAPs were generated from direct nerve stimulation, blue light or CNO delivery. CMAP amplitude, and duration were calculated from each stimulation paradigm. The average stimulation of the sciatic nerve for each paradigm was obtained and averaged across animals.

### RNA isolation and real-time PCR

Muscle tissue was collected from mice at different time points. Tissue RNA was isolated using the Qiagen RNeasy kit for fibrous tissues, according to the manufacturer’s protocol (QIAGEN stock #74704). For real-time PCR, 500 ng of total RNA was DNase I treated (Invitrogen) and reverse transcribed using Superscript II (Invitrogen) reverse transcriptase. A total of 20 ng of cDNA were used in SYBR Green real-time PCR reactions that were performed in duplicate and analyzed on a Step-One real-time PCR machine (Applied Biosystems).

Primer sequences for GAPDH, IL6, TNFα, IL1β, MCP1 were obtained from previously published work [7, 66]. Cycle time (Ct) values for all targets were all normalized to GAPDH as internal control. Differences in expression are determined from the normalized ΔΔCt values and standard error of the difference in means is determined. This was used to calculate fold change between conditions and values are then converted to a percent change where 2-fold = 100% change.

### Statistical analysis

Data were analyzed using GraphPad prism or SigmaPlot software. All values are presented as mean ± SEM unless stated differently. Comparisons of imaging, RT-qPCR data, and electrophysiological responses were tested with a one-way ANOVA, or a two-way repeated measures (RM) ANOVA with Tukey’s post hoc test when appropriate. For behavioral data containing the same animals treated with an intervention over time, a two-way repeated measures (RM) ANOVA was used. Two group comparisons used t-tests and those that failed normality tests were analyzed with a Mann–Whitney U test. The critical significance level was set at p < 0.05. Data will be made available on a public repository upon publication.

## Supplementary Material

Supplement 1
