# Supplementary material for: Synaptic-like coupling of macrophages to myofibers regulates muscle repair
Source: Res Sq. 2024 Oct 28:rs.3.rs-5290399. Preprint. [Version 1] doi: 10.21203/rs.3.rs-5290399/v1 (PMC11581056; doi:10.21203/rs.3.rs-5290399/v1)
Supplement: Supplement 1 [file NIHPPRS5290399V1-supplement-1.pdf]

Supplementary Files

This is a list of supplementary files associated with this preprint. Click to download.

- Tripathietal2024SupplementaryMaterials.pdf
